# A simple technique to identify key recruitment issues in randomised controlled trials: Q-QAT - quanti-qualitative appointment timing

**DOI:** 10.1186/s13063-015-0617-1

**Published:** 2015-03-11

**Authors:** Sangeetha Paramasivan, Sean Strong, Caroline Wilson, Bruce Campbell, Jane M Blazeby, Jenny L Donovan

**Affiliations:** School of Social and Community Medicine, University of Bristol, Canynge Hall, 39 Whatley Road, Bristol, BS8 2PS UK; Royal Devon and Exeter Hospital and University of Exeter Medical School, Exeter, EX2 5DW UK

**Keywords:** Randomised controlled trial, Recruitment to RCTs, Mixed methods, Qualitative research, Quanti-qualitative techniques

## Abstract

**Background:**

Recruitment to pragmatic randomised controlled trials (RCTs) is acknowledged to be difficult, and few interventions have proved to be effective. Previous qualitative research has consistently revealed that recruiters provide imbalanced information about RCT treatments. However, qualitative research can be time-consuming to apply. Within a programme of research to optimise recruitment and informed consent in challenging RCTs, we developed a simple technique, Q-QAT (Quanti-Qualitative Appointment Timing), to systematically investigate and quantify the imbalance to help identify and address recruitment difficulties.

**Methods:**

The Q-QAT technique comprised: 1) quantification of time spent discussing the RCT and its treatments using transcripts of audio-recorded recruitment appointments, 2) targeted qualitative research to understand the obstacles to recruitment and 3) feedback to recruiters on opportunities for improvement. This was applied to two RCTs with different clinical contexts and recruitment processes. Comparisons were made across clinical centres, recruiters and specialties.

**Results:**

In both RCTs, the Q-QAT technique first identified considerable variations in the time spent by recruiters discussing the RCT and its treatments. The patterns emerging from this initial quantification of recruitment appointments then enabled targeted qualitative research to understand the issues and make suggestions to improve recruitment. In RCT1, presentation of the treatments was balanced, but little time was devoted to describing the RCT. Qualitative research revealed patients would have considered participation, but lacked awareness of the RCT. In RCT2, the balance of treatment presentation varied by specialists and centres. Qualitative research revealed difficulties with equipoise and confidence among recruiters presenting the RCT. The quantitative and qualitative findings were well-received by recruiters and opportunities to improve information provision were discussed. A blind coding exercise across three researchers led to the development of guidelines that can be used to apply the Q-QAT technique to other difficult RCTs.

**Conclusion:**

The Q-QAT technique was easy to apply and rapidly identified obstacles to recruitment that could be understood through targeted qualitative research and addressed through feedback. The technique’s combination of quantitative and qualitative findings enabled the presentation of a holistic picture of recruitment challenges and added credibility to the feedback process.

Note: both RCTs in this manuscript asked to be anonymised, so no trial registration details are provided.

## Background

Challenges in recruiting to pragmatic randomised controlled trials (RCTs) evaluating health technologies and services have been well-documented [1-3]. Some strategies to facilitate recruitment through good clinical practice have been identified [4], but systematic reviews have shown that only a small number of interventions to improve recruitment have been robustly evaluated and even fewer are effective [5,6]. A complex intervention using primarily qualitative research methods was developed and applied within the ProtecT (Prostate testing for cancer and Treatment) trial, leading to improved and ultimately successful recruitment [7,8]. An adapted version of this intervention was applied within five further RCTs [9] leading to successful recruitment in some trials [10] and evidence to support a decision to close others [11,12]. A synthesis of the findings that emerged from applying the intervention in these RCTs also provided a detailed understanding of the clear and hidden challenges to recruitment [13] and the fragility of the recruitment process [14]. The intervention comprises methods that can be time-consuming to apply, while RCTs often have time-limited recruitment periods within which to apply interventions; an aspect that has previously proved challenging [11].

There is an urgent need to develop interventions that are simple to apply and can rapidly ensure effective and efficient recruitment of appropriately informed participants to trials. We are undertaking a programme of mixed methods research with two major aims: first to understand recruitment issues as they happen in a wide range and increasing number of pragmatic RCTs [8,9]; and second, to use those nuanced understandings to design components of a complex intervention that could then be assembled to tackle recruitment difficulties in most RCTs. Within this programme, we developed a simple technique - Quanti-Qualitative Appointment Timing (Q-QAT) - to swiftly identify key challenges to recruitment that stem from how the RCT and treatments are portrayed to patients in recruitment appointments (the consultation where RCT information is provided to patients), so that these issues can be fully investigated and suggestions made to improve recruitment. The derivation and development of the technique, its application in two very different RCTs, and some guidance about how it can be applied to RCTs undertaking recruitment, are presented in this paper.

## Methods

### Development of the Q-QAT technique

The principles underlying the Q-QAT technique first emerged in the analysis of audio-recorded recruitment appointments in the ProtecT trial feasibility study. It was noted that one among the key factors inhibiting recruitment in this study was the lack of balance in the presentation of the treatments to patients. For instance, recruiters presented the trial treatments in a particular order (surgery, radiotherapy and, finally, monitoring), at different lengths and with different degrees of enthusiasm (monitoring presented more briefly and with less enthusiasm than the other two treatments), meaning that very few potential participants were willing to be randomised [7]. When recruiters were given suggestions to correct this imbalanced presentation of treatments (among other aspects of the complex intervention) [7], recruitment improved [8]. As similar imbalances in the presentation of RCT treatments were also found in other RCTs [10,11], we set out to develop a simple technique, Q-QAT, with three main aims: a) to systematically investigate, quantify and qualitatively explore these imbalances, b) to assess the practicality of the Q-QAT technique, its acceptability amongst recruiters, and its potential usefulness as an intervention to improve recruitment and c) to develop guidelines to allow easy future application of the technique.

### Application of the Q-QAT technique in two RCTs

The Q-QAT technique was further developed and standardised within two RCTs.

#### Setting - the RCTs

The two RCTs were pragmatic, publicly-funded studies that differed in terms of organisational issues and complexity of the recruitment process so that they would test the technique (Table 1). In RCT1, there was one specialty (surgery) involved, it compared surgery and a minimally invasive technique to treat an elective problem, and had a relatively simple recruitment process comprising an appointment with a surgeon where the trial was discussed, with a face-to-face or telephone follow-up by a research nurse. In contrast, RCT2 involved two clinical specialties (surgery and oncology), compared surgery and radiotherapy to treat a life-threatening cancer (all patients had chemotherapy first), and had a more complex recruitment process in which patients had separate appointments with each specialist before being asked to consider participation in the RCT. In both RCTs, patients were given the trial information sheet at the appointments to take home and consider. The research nurse usually called after a week to elicit the patient’s decision.

**Table 1:** **Characteristics of the randomised controlled trials (RCTs) used to develop the Quanti-Qualitative Appointment Timing (Q-QAT) technique**

	**RCT1**	**RCT2**
Context	Elective procedure, not life-threatening	Life-threatening cancer
Specialties	Surgery	Surgery and oncology
Treatment groups	Standard surgery versus minimally invasive procedure	Chemotherapy plus either surgery or radiotherapy
Centres for qualitative research	1	3
Clinicians who provided audio-recordings	3 surgeons; 1 research nurse	Centre 1: 4 surgeons, 2 oncologists
		Centre 2: 1 surgeon, 2 oncologists
		Centre 3: none
Recruitment process	Treatment options and recruitment discussion by surgeon, in the same appointment as history-taking, diagnosis and examination. Sometimes follow-up by nurse	Dedicated appointments for treatment options and recruitment discussion. Separate appointments with surgeon and with oncologist
Number of consultation audio-recordings obtained	13 with surgeons	Total: 26 pairs
	8 with research nurse	(Centre 1: 19 pairs, Centre 2: 7 pairs, Centre 3: none)
Q-QAT applied to	13 surgeon appointments	Total: 11 pairs
		(Centre 1: 7 pairs, Centre 2: 4 pairs)
Number of interviews	5 patient interviews	18 interviews with 16 patients
	Unrecorded discussions with CI	(Centre 1: 9, Centre 2: 3, Centre 3: 4)
		20 staff interviews
		(Centre 1: 10, Centre 2: 4, Centre 3: 6)
		Unrecorded discussions with CI

*Abbreviation*: *CI* Chief Investigator.

The RCTs were also at different stages, and with different degrees of collaboration with the Q-QAT team. In RCT1, recruitment to the full trial was underway, but, with concerns about low levels of recruitment, the CI permitted a small nested qualitative study in one centre to investigate this. In RCT2, which was a feasibility study, there was a fully-funded integrated qualitative study modelled on previous qualitative research in trials applied to all three centres [8]. Ethical approval was obtained for the qualitative study as an amendment for RCT1, and integrated into the approval for RCT2. The names of the approving bodies cannot be provided because the two RCTs asked to be anonymised.

#### Data collection

Informed consent was obtained from patients and RCT staff prior to the audio-recording of consecutive recruitment appointments and in-depth interviews. Digital recorders were delivered to participating centres with instructions for use to facilitate audio-recording of recruitment appointments. In-depth interviews were conducted with a purposive sample of patients in both RCTs as well as staff in RCT2, using a semi-structured approach and topic guides based on previous studies [8,9]. In both RCTs, patients were interviewed after their recruitment appointment and after they had made a decision on trial participation. They were asked about their views on the information provided at the recruitment appointment and the key issues that influenced their decision-making process in relation to trial participation. In RCT2, staff interviews were conducted during recruitment and participants were asked about their views on the RCT and its treatments, challenges to recruitment and ways to overcome them. Interviews with staff were not undertaken in RCT1 because of resource constraints. Interviews, Q-QAT application and analysis in RCT1 were carried out by SP and in RCT2 by SS and CW.

#### Data analysis: Q-QAT elements

Recruitment-related parts of the audio-recordings of recruitment appointments and all parts of the interviews were transcribed. The Q-QAT technique was then applied to eligible patients’ recordings in three ways, each of which is described in detail below.

1. Q-QAT quantification: the following aspects were coded and quantified in transcripts of recruitment appointments:
  - Time taken by recruiters to present the details of each of the RCT treatments (mean, median, range; by centre, recruiter, specialty as applicable)
  - Time taken by recruiters to explain the design, purpose and procedures of the RCT (mean, median, range; by centre, recruiter, specialty as applicable)
  - Total length of appointment

2. Targeted qualitative analysis: the findings from the Q-QAT quantification process informed the targeted exploration of issues during the qualitative analysis. The recruitment appointments and interviews were thematically analysed using constant comparative techniques from grounded theory [15], drawing on methods that had been used in similar previous studies [8,9].
  - Recruitment appointments were analysed to understand the barriers to recruitment in appointments
  - Interviews were analysed to understand the views of recruiters and/or patients about the RCT and recruitment difficulties, and to assess the acceptability and usefulness of the Q-QAT technique with the RCT Chief Investigator (CI), centre Principal Investigators (PIs) and recruiters

3. Feedback: findings from the analysis in 1 and 2 above were synthesised and discussed with the RCT CI in the first instance. Presentations were then made to groups of recruiters in face-to-face meetings informing them of the anonymised findings and identifying opportunities to improve recruitment.

These methods were investigated first in RCT1, which had a simpler recruitment process, and then applied and further refined in RCT2, which was more complex.

### Development of guidelines for future application of the Q-QAT technique

**Figure 1:**
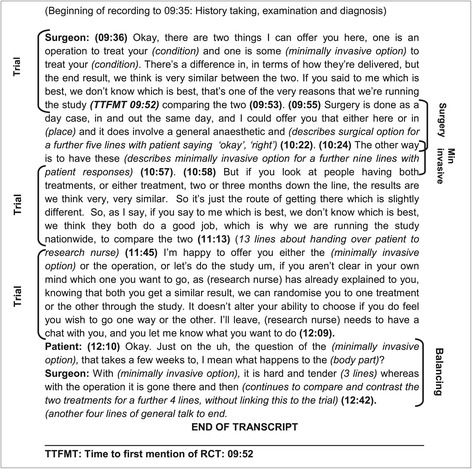
**Example of transcript with Quanti-Qualitative Appointment Timing (Q-QAT) coding from randomised controlled trial 1 (RCT1).** Transcripts were coded with time markings that denoted when a particular code began and ended. An example of how this was done is shown here.

In order to refine and standardise the technique for future use, 3 researchers (SP, SS and CW) separately coded a sub-sample of 6 appointments (2 in RCT1 and 4 in RCT2) and then compared the codes to develop the consistency and reliability of the Q-QAT coding strategy. During this process of blind coding, in addition to the aspects of time identified above (time taken to present the RCT treatments and the RCT), other key aspects of recruitment (such as balancing of treatments) were identified and assessed for their importance and ability to be added to the Q-QAT technique. Coding challenges such as overlapping topics of conversation and quick comparisons of treatments where recruiters frequently moved from one treatment to another were specifically considered. Regular meetings were held amongst the researchers to refine the codes, resolve discrepancies and develop guidelines for the use of the Q-QAT technique. The final version of the guidelines is provided in detail below and an example of a transcript with Q-QAT coding is provided in Figure 1.

## Results

### Application of the Q-QAT technique in RCT1 and RCT2

In RCT1, there were 13 audio-recordings of recruitment appointments with surgeons in 1 centre and in RCT2, 26 pairs of recruitment appointments, each with an oncologist and surgeon in 2 centres (Table 1). Recruitment to the RCTs had been very limited at the time of Q-QAT application. All recordings during the Q-QAT study were with eligible patients. The Q-QAT technique was applied to all 13 appointments in RCT1; in RCT2, it was applied to the first 11 pairs of recordings to be able to provide rapid feedback to recruiters.

In RCT1, there were 8 follow-up appointments with the research nurse and 5 interviews with patients, and in RCT2, there were 20 interviews with recruitment staff and 18 with patients. The findings are presented below for each RCT separately.

#### Findings from RCT1

1. Q-QAT quantification

Three surgeons provided at least 4 recordings each in RCT1 (Table 2). The appointments ranged in length from around 9 to 29 minutes (mean 17:02; median 16:22).

**Table 2:** **Randomised controlled trial 1 (RCT1) Quanti-Qualitative Appointment Timing (Q-QAT) timings in minutes: seconds**

**Patients**	**Recruiters**	**Total time**	**Surgery**	**Minimally invasive procedure**	**RCT**
Patient 1	Surgeon X	12:54	00:35	00:48	00:56
Patient 2	Surgeon X	16:22	00:29	00:13	00:40
Patient 3	Surgeon X	18:51	01:13	01:10	00:28
Patient 4	Surgeon X	29:14	00:44	00:59	01:46
Patient 5	Surgeon X	19:59	00:43	01:45	01:06
Patient 6	Surgeon Y	11:28	00:53	00:52	01:42
Patient 7	Surgeon Y	17:43	00:31	01:29	03:08
Patient 8	Surgeon Y	12:46	00:48	01:08	01:35
Patient 9	Surgeon Y	15:33	00:32	01:03	02:09
Patient 10	Surgeon Z	12:15	00:00	05:02	02:32
Patient 11	Surgeon Z	08:58	02:00	02:46	02:10
Patient 12	Surgeon Z	24:16	02:20	05:06	02:39
Patient 13	Surgeon Z	21:09	00:56	02:13	03:03
Mean		17:02	00:54	01:54	01:50
Median		16:22	00:44	01:10	01:46
Range		08:58 to 29:14	00:00 to 02:20	00:13 to 05:06	00:28 to 03:08

The Q-QAT table in RCT1 shows the time breakdown for a total of 13 recordings with 3 surgeons. The total time of the recording and the time spent on describing the treatments (surgery and minimally invasive procedure) and the RCT were recorded. The remainder of the time was spent on other aspects such as history-taking, examination, diagnosis and non-RCT related treatment information.

- Time taken by recruiters to present the details of each of the RCT treatments: the description of surgery ranged from ‘no discernible discussion’ to around 2 minutes (mean 00:54, median 00:44). The description of the minimally invasive technique ranged from 13 seconds to around 5 minutes (mean 01:54, median 01:10). Surgeons, on average, spent a little more time discussing the minimally invasive technique than surgery, although this imbalance was most evident in 6 of the 13 appointments (appointments 5, 7, 9, 10, 12 and 13).
- Time taken by recruiters to explain the design, purpose and procedures of the RCT: discussion about the RCT ranged from 28 seconds to just over 3 minutes (mean 1:50, median 1:46). Comparing recruitment rates and time spent on the RCT, surgeon X, with the lowest recruitment rate (7%, 13/183) spent the least amount of time explaining the RCT (mean 01:14), whereas surgeon Z, with the highest recruitment rate (19%, 19/99) spent the greatest amount of time discussing the RCT (mean 02:36). Surgeon Y was between the others on both counts (RCT mean 02:09; recruitment rate 13.6%, 19/140).

In summary, some preliminary conclusions and questions worthy of further investigation could be drawn from the simple Q-QAT quantification above in RCT1. First, the reasons why surgeons spent a little more time, on average, describing the minimally invasive technique, and why this was more marked in some appointments than others, could be explored. Second, very little time was spent discussing either the RCT or its treatments, compared with other information or activity in the appointments, and the impact of this on recruitment was identified as important to investigate. Also, although there were only a small number of recordings, there seemed to be a relationship between time spent explaining the RCT and recruitment success. Each of these issues was explored in the qualitative analysis below.

2. Targeted qualitative analysis
  - Insights from recruitment appointments

The qualitative analysis revealed that the reason for less time devoted to surgery, identified by the Q-QAT analysis in some appointments, was because surgeons provided minimal information for patients with previous experience of the procedure (appointments 5, 10, 13) and used the time to explain the recent advances in minimally invasive treatment (appointment 9). In 7 of the 13 appointments, there was a good time balance between treatments, which reflected the way in which the treatments were portrayed:

*Surgeon Z: there are good things about T(a); there are good things about T(b).*

*Surgeon Y: we know they both work perfectly well, it’s just that there’s different things to them. On the one hand, one has an anaesthetic, the other doesn’t. One, uh, you know, you’ve got to wear some bandages for longer, one you are left with* (problem) *for longer, but you haven’t had an anaesthetic, and there’s all these pros and cons, and we really don’t know whether one is better than the other*.

This kind of balanced information about treatments prepared patients to be recruited to the RCT, but the qualitative analysis confirmed that the majority of time in RCT1 appointments was spent on history-taking, examination, diagnosis and non-RCT related treatment information, with very little being said about the RCT itself. Surgeons tended to present the RCT briefly and awkwardly, sometimes as an option only for patients who did not have a treatment preference:

*Surgeon X: if you didn’t decide* (on the treatment) *or couldn’t decide which way you’d like to go with your choice, or you’re happy to leave your choice open to us, we are randomising people*.

When they did mention the RCT, they struggled to present it clearly and complete recruitment. In the appointment below, the patient repeatedly indicated her equipoise by stating she had *‘no favourite’* treatment, was *‘ambivalent’* and expressed a positive attitude towards the study and the 5-year follow-up provided. However, she was asked to consider the study only if she could not make her mind up, with a subtle remark that she may think the study was ‘*too much bother’* (patient’s interpretation of this discussion is presented in section 2(b) below):

*Surgeon Z: and if you go into the study, it would involve us opening an envelope at random so to speak and it will say treatment (a) or treatment (b). We then do the treatment and follow you up afterwards for 5 years to make sure.*

*Patient 11: yeah, that’s interesting.*

*Surgeon Z: it won’t involve a lot of visits or extra time* (explains follow-up questionnaires for five lines)*. So the most important thing is that if you decide you’d rather have treatment (a) or you’d rather have treatment (b)…*

*Patient 11: I’m pretty ambivalent really.*

*Surgeon Z: yes, well just don’t make that decision in front of me today. And if you can’t make your mind up, consider the study, but if you don’t want to do the study ‘cause you’re thinking it might be too much bother, you don’t have to give us a reason, it will not alter the way we feel about you in anyway. It’s simple, you can walk away at any time.*

*Patient 11: excellent.*

Exploring the relationship between time spent discussing the RCT and recruitment success across surgeons was difficult because no patients had agreed to participate in the RCT during the time of Q-QAT analysis. However, the recordings of patients who declined revealed that the extra time spent by surgeon Z provided the scope to address questions, explain the study positively and in detail, including aspects of follow-up and benefits of study participation, which were discussed to a far lesser extent or not at all by the other surgeons.

The qualitative analysis also raised questions about who discussed the RCT in detail. Following their limited discussion or mention of the RCT, the surgeons often ended the appointments by suggesting that the patient should discuss the issues further with the research nurse involved in the study. The conversations with the nurse (usually on the telephone), however, were very short and focused only on patients’ travel plans, appointment arrangements, and their decision about choice of treatment rather than any discussion about trial participation:

*Research Nurse (RN):* after initial introductions) *It’s just a follow-up call to see if you’ve had a chance to read through the information you were given about the treatment options.*

*P8: I have yes.*

*RN: yes. What would you like to do?*

*P8: what I’ve decided I wanna do is, if possible is to have the operation if that’s alright.*

*RN: okay, yes. I’ll let* (surgeon’s) *secretary know that.*

(b) Insights from interviews with patients

Interviews with patients confirmed the consequences of the Q-QAT findings. Because very little time was spent discussing the RCT, patients had little if any understanding of what trial participation or randomisation involved. Two patients who wanted to participate in the study because they found the long-term follow-up offered quite appealing, thought they could choose a treatment and still be in the RCT. One of them, patient 11 from above, reflected on the appointment and the decision to choose a treatment rather than enrolling in the RCT:

*Patient 11: he* (surgeon) *said you have to choose whether you want to have treatment (a) or whether you want to have treatment (b). And he explained them both and really they didn’t seem to make any difference. He seemed to think that at my age and my health that it wasn’t a problem having an anaesthetic, and I said well, I really am ambivalent. He said ‘no’, go home and read the paperwork and think about it. And don’t make a decision today. So I did that, I went home and then I decided I would go for treatment (b)*.

Later, on explaining the concept of randomisation during the interview, this patient clearly stated willingness to participate in the trial even if allocated to treatment (a). She explained that she chose a treatment because the surgeon said *‘you have to make a decision’*, which was taken to mean a decision on which treatment she wanted to have rather than on whether she wanted to participate in the trial or not.

The interviews indicated other missed opportunities for recruitment such as patients who did not have a strong treatment preference prior to or during the recruitment appointment - a reflection of the reasonably well-balanced presentation of information as shown in Q-QAT and an indication that these patients were in equipoise and, therefore, ideal candidates for randomisation. However, similar to patient 11 above, these patients later chose a treatment because they thought that was the decision being asked of them and also because they could not see the link between their uncertainty about which treatment to have and the rationale for the trial:

*SP: did you have any opinion as to what treatment you might prefer before you went in for this initial consultation?*

*Patient 4: no, I didn’t know anything about it to be quite honest.*

*SP: so when did the fact that you preferred surgery kind of take shape do you think? During the consultation, after or?*

*Patient 4: well, I came home and they sent me information or I had information about both of them, I think it was just reading that really, because at the consultation* (surgeon) *couldn’t, he explained both the treatments but he didn’t, I was disappointed that he couldn’t turn around and say to me, treatment (a) would be better for you or treatment (b) would be better for you. So although he explained both procedures to me, I didn’t really choose there and then what I was gonna have.*

*SP: and did* (surgeon) *kind of explain why he was not able to choose one treatment for you?*

*Patient 4: I don’t know, I can’t remember what he said, why he couldn’t, but I remember asking him but I can’t think what he said.*

A few patients understood some aspects of randomisation, but without being provided with a clear rationale for it, found the concept confusing or misunderstood its purpose as solely the generation of equal-numbered groups:

*Patient 4: because I was eligible for both of them, I would benefit from either of the treatments, he said to me, and it would just be almost picked out of a hat.*

*SP: was there any reason do you think for the treatment being chosen in that way?*

*Patient 4: um, I’m not sure really. I don’t know.*

*Patient 14: had there been an option where equal numbers of people said this was the way they wanted to go, I don’t see why they couldn’t have been in the trial, you know, they could have still been followed up, they could have still been kept under scrutiny and the results would still have been the same because there were the same number of people.*

3. Discussion of the Q-QAT and qualitative findings with RCT1 CI, PI and recruiters

The Q-QAT analysis in RCT1 showed that recruiters provided a reasonably well-balanced presentation of treatments, but spent very little time explaining the RCT. The qualitative analysis emphasised that the limited time spent explaining the RCT did not enable sufficient information provision for patients to consider the RCT properly. It also showed that the surgeons were finding it difficult to present the RCT with clarity. These findings were reflected in the patient interviews, which showed that patients had potentially been in equipoise before, during and for a brief period after the consultation, but their uncertainty was not then linked to trial participation or randomisation, and so patients thought they had to choose a treatment. In addition, the research nurse was expected to assist with recruitment, but seemed not to be aware of this and did not do so.

The findings were discussed with the CI and then presented to the centre PI and recruiters in other trial centres. A short document was produced to provide feedback for recruiters to assist them in providing clearer information about the RCT and randomisation. The nurse was encouraged to engage in a more in-depth patient-led discussion of what RCT participation involved. At the feedback meetings, recruiters found the Q-QAT analysis to be a useful tool to visualise the time breakdown of their appointments and felt that the qualitative insights helped explain the Q-QAT findings further. While resources were not available to support a full evaluation, feedback from recruiters suggested that recruitment improved as a result of the information provided during feedback.

#### Findings from RCT2

In RCT2, there was a more complex recruitment pathway where patients with the cancer diagnosis had appointments with both a surgeon and an oncologist to explain the treatment options and the RCT. All 3 centres agreed to participate in the qualitative recruitment study, although only 2 actually provided audio-recorded appointments - 26 pairs in total of which Q-QAT was applied to the first 11 pairs to facilitate early feedback provision, 7 in Centre 1 and 4 in Centre 2 (Tables 3 and 4). Q-QAT analysis was carried out separately by centre and specialty.

**Table 3:** **Randomised controlled trial 2 (RCT2) Quanti-Qualitative Appointment Timing (Q-QAT) timings in minutes: seconds, Centre 1**

**Patients**	**Recruiters**	**Total time**	**Surgery**	**Radiotherapy**	**Trial**
Patient 1	Surgeon A	28:54	02:41	03:01	08:01
	Oncologist A	37:34	00:10	08:48	03:15
Patient 2	Surgeon B	35:27	02:23	02:05	03:20
	Oncologist B	15:31	00:10	00:41	00:00
Patient 3	Surgeon C	41:00	03:00	01:59	05:44
	Oncologist B	25:30	00:10	04:59	01:20
Patient 4	Surgeon C	17:30	00:30	00:15	01:30
	Oncologist B	12:52	00:34	02:15	00:40
Patient 5	Surgeon B	42:05	00:25	01:20	05:45
	Oncologist B	20:07	00:00	03:23	00:30
Patient 6	Surgeon A	12:59	02:24	00:10	00:50
	Oncologist B	09:14	00:50	00:10	00:00
Patient 7	Surgeon D	18:11	01:05	00:00	04:06
	Oncologist B	33:08	00:10	04:30	01:23
Centre 1 Total	Mean	25:00	01:02	02:24	02:36
	Median	22:49	00:32	02:02	01:26
	Range	09:14 to 42:05	00:00 to 03:00	00:00 to 08:48	00:00 to 08:01
Centre 1 Surgeons	Mean	28:01	02:32	01:16	04:11
	Median	28:54	02:23	01:20	04:06
	Range	12:59 to 42:05	00:25 to 03:00	00:00 to 03:01	00:50 to 08:01
Centre 1 Oncologists	Mean	22:00	00:18	03:32	01:01
	Median	20:07	00:10	03:23	00:40
	Range	09:14 to 37:34	00:00 to 00:50	00:10 to 08:48	00:00 to 03:15

**Table 4:** **Randomised controlled trial 2 (RCT2) recruiter Quanti-Qualitative Appointment Timing (Q-QAT) timings in minutes: seconds, Centre 2**

**Patients**	**Recruiters**	**Total time**	**Surgery**	**Radiotherapy**	**Trial**
Patient 8	Surgeon E	31:00	04:50	01.15	02:55
	Oncologist C	38:02	00:10	12:38	02:00
Patient 9	Surgeon E	28:02	03:11	01:57	05:35
	Oncologist C	31:00	00:15	14:14	01:05
Patient 10	Surgeon E	38:10	03:20	00:00	03:32
	Oncologist C	33:31	00:15	00:00	00:00
Patient 11	Surgeon E	28:02	01:19	00:20	01:05
	Oncologist D	33:46	01:26	00:52	02:49
Centre 2 Total	Mean	32:42	01:51	03:55	02:23
	Median	34:31	01:23	01:04	02:25
	Range	28:02 to 38:10	00:10 to 04:50	00:00 to 14:14	00:00 to 05:35
Centre 2 Surgeon	Mean	31:19	03:10	00:53	03:17
	Median	29:31	03:16	00:48	03:14
	Range	28:02 to 38:10	01:19 to 04:50	00:00 to 01:57	01:05 to 05:35
Centre 2 Oncologists	Mean	34:05	00:32	06:56	01:29
	Median	33:39	00:15	06:45	01:33
	Range	31:00 to 38:02	00:10 to 01:26	00:00 to 14:14	00:00 to 02:49

1. Q-QAT quantification

The appointments in RCT2 ranged in length from around 9 to 40 minutes. On average, appointments took around 30 minutes across both centres - slightly longer in Centre 2 than Centre 1. The RCT and its treatments took up a relatively small part of these appointments.

- Time taken by recruiters to present the details of each of the RCT treatments: in both centres, on average, less time was spent on surgery than radiotherapy. However, the time spent describing each treatment varied by specialty in both centres. On average, surgeons spent more time describing surgery than radiotherapy and oncologists spent more time describing radiotherapy than surgery - and this was consistent across both centres. The time imbalance appeared more prominent among oncologists than among surgeons.
- Time taken by recruiters to explain the design, purpose and procedures of the RCT: on average, a similar, relatively short time was spent in both centres by all specialists on explaining the RCT. The Q-QAT analysis indicated that surgeons spent more time discussing the RCT than oncologists in both centres.

As in RCT 1, some preliminary conclusions and areas of further investigation could be drawn from the simple Q-QAT analysis in RCT2. Recruitment in this RCT was very difficult - no patients had been recruited (of 14 eligible patients) across the 3 centres at the time of Q-QAT analysis. Issues to investigate qualitatively included whether imbalances in the presentation of treatment and RCT information by the different specialists was having any influence on recruitment and whether the time taken to explain the RCT was sufficient to enable recruitment. Each of these issues was explored.

2. Targeted qualitative analysis
  - Insights from appointments

Analysis of all 26 paired recordings (19 in Centre 1; 7 in Centre 2) of patient recruitment appointments with both oncologists and surgeons showed that although specialists spent more time discussing their own treatment as shown by Q-QAT, in general, specialists across both centres unwittingly tended to convey a subtle bias towards surgery despite acknowledging that they did not know which treatment was better. Surgery was described using definitive terms such as ‘cure’, ‘kills the cancer’, ‘gold standard’, ‘physically removes’ or ‘cuts it away’. Both sets of specialists then tended to reinforce this by expressing doubts about the effectiveness of radiotherapy and described it tentatively as having the ‘chance of killing the tumour’, ‘tries to kill it’, ‘shrinks the tumour’, ‘may be able to treat the tumour’ or as an option for those who did not want an operation:

*Oncologist B, Centre 1: it’s a very difficult decision and there isn’t a right and a wrong answer and some people have very clear ideas about what they want to do, whether it should be an operation, they want to have it cut out. Other people, the thought of the operation is just so frightening they opt for the radiotherapy treatment. But for us, we don’t know which of these treatments is - is better.*

*Oncologist C, Centre 2: getting back to what you said before about eradicating it* (tumour) *was your word I think, the problem if you think about it with, and especially in radiotherapy is partly that we do the treatment and then when you finish you have a scan. You can still see where the tumour is or where it’s been ‘cause it’s, then takes time for it to shrink and as it shrinks, the area that was treated becomes inflamed and then scarred and you look at the scans after this sort of treatment and you’re always looking at it and thinking, ‘is there still tumour there or is there not?’ and it’s difficult sometimes to assess response after radiotherapy* (three more lines about assessing the scans)*. Whereas at least with surgery, as you say, once it’s cut out, that’s the end of it hopefully and then the scans that we do are slightly different - they’re to look to see if there’s any sign of it coming back and you have scans in the future, er, er, yeah, so it is, some people do feel that that they want to have it cut out and that - they feel that that’s gonna be the end of it.*

*Surgeon E, Centre 2: now, the upside of the chemotherapy and the radiotherapy is of course you don’t need a very big operation. The upside of surgery is that of course we actually physically remove the cancerous area and we are able to look at it under the microscope and see exactly what the nature of it is and, and so on, but the evidence that we have is that the two techniques are similar, but we don’t know whether they’re exactly the same and that is why we’re doing the study.*

The same surgeon later continued as follows:

*Surgeon E, Centre 2: the chemotherapy sensitises the tumour and then the radiotherapy blasts it and tries to- to- to- kill it. And there have been some very good results when we do this form of treatment but they’ve never ever been compared with what has been in - over many years, the gold standard treatment which is, with, with an operation*

The presentation of information about the RCT was often very short, with minimal detail, and was sometimes hesitant and unclear:

*Patient 2: I mean, what is the trial? That’s what I’m trying to get at.*

*Surgeon B, Centre 1: the trial is, we would enter you into this trial and the trial would choose the option for you.*

*Patient 2: does the trial, is it something else that’s going to start before we actually get the treatment?*

*Surgeon B: no. Er, n- n- n- not quite. The treatment is either chemotherapy and surgery or chemotherapy and radiotherapy, and if you agree to enter this trial, we would choose - well, the study would choose one of those options for you, at random.*

*Patient 2: yeah, but what have I got to do for the trial, this is the point, is what I’m trying to get at.*

*Surgeon B: you would have that treatment, whichever one the trial chose for you.*

*Patient 2: no, I don’t think I’m getting this. This word trial to me seems to be a completely wrong word. It’s not a trial, it’s actually what you’re gonna get. It’s the actual treatment, isn’t it?*

(b) Insights from interviews with recruitment staff and patients

Interviews were undertaken with 20 staff and 16 patients. In their interviews, specialists also used phrases similar to those in their recruitment consultations to describe the treatments (surgery ‘*cuts out’, ‘physically removes’* versus radiotherapy *‘shrinks’, ‘sterilises’ ‘keeps the tumour’*). A few showed awareness of their biases and acknowledged the impact of this language and of an imbalanced presentation of the treatments on recruitment:

*Surgeon G, Centre 1: we can certainly stir up and reinforce a patient’s bias very easily with a throw away comment like the aim of the surgery is to cut out your cancer and that could ruin everything from the point of view of a balanced randomised trial.*

*Oncologist B, Centre 1: there was someone who used to work here who used to say ‘well I can cut away your tumour or they can shrink it with radiotherapy’, so the word you use matters a lot because ‘shrink’ gives the impression that you’re not going to get rid of it, whereas to cut it out means you’ve taken it away, gives you the impression that you’re definitely cured.*

In Centre 2, some of the surgical bias was attributed to surgery being considered the *‘gold standard’* in the centre prior to the trial:

*Oncologist E, Centre 2: it is the case though that historically because we’ve had such a strong surgical lead to the MDT* (Multi-Disciplinary Team meeting)*, we’ve got a long history of surgery for that group of patients* (*…*) *I think that it’s not because we think surgery is better, it’s just that we’ve more experience and that it’s been a gold standard here for so long.*

When interviewed, patients’ views on the treatments generally reflected specialists’ subtle bias towards surgery and they tended to mirror the language used by the specialists:

*Patient 14, Centre 1: so having chemo and radiotherapy, there’s no guarantee with any of it, but in my mind, you’re just trying to shrink the tumour and there is no guarantee that you will do it with that, but with surgery you’re physically cutting it out.*

Centre 1 staff interviews revealed that these biases were more readily and strongly attributed to surgeons, by themselves and by oncologists, with only infrequent acknowledgement of oncologists’ biases and preferences. In contrast, although patients generally tended to say they did not think that either of the specialists had a preference, some patients felt they detected a preference for radiotherapy among oncologists because they spent more time explaining radiotherapy:

*Patient 4, Centre 1: once or twice I thought is he* (oncologist) *trying to persuade you to go for the radiotherapy and chemotherapy, that was the impression I got, but I don’t know. (CW: what did he say that made you think that?). Patient 4: well he sort of elaborated on it a bit more rather than about the operation. I had a feeling he was leaning towards that way. (CW: and when you were with* (surgeon) *did you feel was leaning towards the surgery?) Patient 4: no,* (surgeon) *explained what surgery would be, what radiotherapy would be, and said really the decision will be yours.*

In both centres, clinicians’ time and content imbalances in explaining the treatments (Tables 3 and 4) had consequences as many patients clearly grasped the competing interests of the two clinicians they saw:

*Patient 2, Centre 1: my honest opinion was between them there was a little bit of competition, and that can put you off a little bit. There shouldn’t be competition in that sort of a way (…) they might not think so themselves but there is, yeah. Obviously the surgeon thinks he’s the better man, the other one thinks he’s the better man.*

*Patient 14, Centre 1: I know that there is historically an antipathy between oncology and surgery, different aspects of looking at things.*

*Patient 17, Centre 2: I got the impression, and you can take it whichever way you like, that they knew what they were doing with their job and the other, they weren’t quite interested in the other side, if you know what I mean.*

Interviews with patients showed that they had very little knowledge or understanding of the RCT, and confirmed that discussion about randomisation was absent or very minimal in the recruitment appointments they had. In Centre 3 staff interviews and meetings, the oncologists admitted being sceptical about whether the trial could be carried out successfully from its outset and readily questioned surgeons’ commitment to the trial.

3. Discussion of the Q-QAT and qualitative findings with the RCT CI

In RCT2, the Q-QAT analysis identified two potential barriers to recruitment: unequal time spent describing treatments and very little time spent explaining the RCT. When these were explored further in the qualitative analysis, they were confirmed as key barriers. There was also evidence of a lack of equipoise in Centres 1 and 2 among specialists, with a tendency to prefer surgery. In interviews with specialists, some acknowledged this bias, its origins and consequences for recruitment, although most of this discussion was centred on surgeons being more biased than oncologists. However, the evidence from the Q-QAT findings did not support this because there were imbalances in information provision across both specialties.

Patients’ views about treatments reflected those of the specialists. Although patients tended to say that the specialists did not appear biased, they sensed the competition between the two specialties. In Centre 3, it appeared that there were problems with oncologists’ belief in the trial. In general, considerable discomfort was evident among the specialists in the very short and sometimes confusing descriptions of the RCT design and purpose, and inevitably patients’ understanding of the RCT was very limited.

These findings were discussed with the CI. As Q-QAT was applied as part of a fully-funded integrated qualitative recruitment study in RCT2, anonymised findings were then presented at an open meeting to 16 recruiters, including oncologists, surgeons and representatives from all 3 centres. The presentation of Q-QAT tables and the targeted qualitative analysis provoked considerable discussion amongst recruiters. During the meeting, recruiters from Centre 3 admitted that they did not have sufficient equipoise and belief in the RCT to take part in it, and they did not recruit further. Recruiters from Centres 1 and 2 believed they could achieve equipoise. Recruiters in Centre 2 approached 10 patients over the following 15 months, but they continued to favour surgery and so no patients agreed to join RCT2. In Centre 1, recruiters approached 16 eligible patients during this time, and, in more balanced appointments, 5 patents agreed to be randomised. It was not possible for RCT2 to recruit sufficient numbers for a full trial.

### Guidelines for applying the Q-QAT method

For the Q-QAT method to be practicably useful, it was tested for consistency and reliability by 3 researchers (SP, SS and CW) coding the appointments in RCTs1 and 2 independently and then comparing coding outcomes so that guidance could be prepared for future use. The final refined coding strategy is presented below. It comprises six main codes - the original three (a to c) and three additional codes (d to f) that emerged as a result of the blind coding process. Each of the six codes is presented below with a detailed description of what they should contain and guidance on the interpretation of patterns. Figure 1 provides an example of the final coding process with the start and finish times of examples of the Q-QAT data items and Table 5 gives a brief outline of the coding categories.

**Table 5:** **Quanti-Qualitative Appointment Timing (Q-QAT) coding categories**

**Coding categories**	**Content**
(a) Trial group 1, Trial group 2 and so on	Time spent by recruiter and patient discussing:
	Intervention types
	Intervention processes
	Intervention outcomes
	(Content of each RCT treatment group to be defined for each randomised controlled trial (RCT))
(b) Trial	Time spent by recruiter and patient discussing:
	RCT design
	Rationale for RCT
	Patient eligibility
	Processes involved in trial participation, including randomisation, informed consent, study documentation and procedures
(c) Total length of appointment	Time spent by recruiter and patient discussing:
	Everything - from start to finish of appointment
(d) Balancing	Time spent by recruiter and patient discussing:
	Need for intervention when not in context of RCT
	All other intervention options available when not in context of RCT
	Eligibility for any treatment when not in context of RCT
	Discussion of interventions involving comparisons, when not in the context of the RCT
(e) TTFMT	Time elapsed before first mention of RCT
(Time to first mention of RCT)	
(f) Other	Time spent by recruiter and patient discussing:
	All other issues, including current state of health, history-taking,
	test results, diagnosis, examination, and general non-RCT or
	intervention talk

#### Q-QAT coding framework

- RCT treatment groups: discussion of intervention types, processes and outcomes related to each RCT treatment. *Codes: name of Trial group 1, Trial group 2,* and so on*.* Imbalance in time spent between treatments or specialists could suggest underlying issues with equipoise or preferences for particular treatments.
- RCT design and processes: all aspects of the presentation of the RCT, including the RCT design and rationale, eligibility, clinical uncertainty/equipoise, and processes involved in RCT participation, such as randomisation, informed consent, follow-up schedules. *Code: trial.* Spending little time discussing these issues could suggest discomfort with the RCT, a lack of integration of the RCT into clinical practice or unfamiliarity with recruitment.
- Total length of the appointment: the entire appointment length, from start to finish. *Code: total time.* This is useful to calculate the proportion of time spent discussing the trial and/or the treatments in relation to the whole appointment.
- Balancing of treatments: comparisons of the advantages, disadvantages and evidence of the RCT treatments, expressions of uncertainty, justifications for the need for treatment, and suitability for treatments not included in the RCT. *Code: balancing.* This was added to capture crucial aspects of the discussion that are nonetheless not directly linked to the RCT by the recruiters or when not already included in codes (a) and (b) above. It was also added to overcome the challenge of coding sections where the recruiter constantly moved from one treatment to another such that each line or turn in the conversation cannot be accurately timed. This code is to be used with codes (a) and (b) to indicate the level of integration of the RCT in the appointment, and willingness to compare intervention options.
- Time to the first mention of the RCT (TTFMT): the length of time that elapsed before the RCT was mentioned. *Code: TTFMT.* This was added to give an indication of how much the RCT was integrated into or framed the overall discussion of the appointment.
- All other matters discussed: all other aspects discussed in the consultation. *Code: other.* This was added to indicate the proportion of the discussion dedicated to trial recruitment (a, b, d) in comparison to other aspects, such as, history-taking, examinations and discussion of other issues.

#### Coding guidelines

The above codes need to be adapted to suit the particular RCT design in terms of the number and type of interventions available. Transcripts or audio-recordings of appointments must be available. Recruiter and patient contributions to the Q-QAT data items should be included in the coded timings to capture the time spent ‘discussing’ them. Codes can then be applied systematically and raw times (and means, medians and ranges) calculated to produce the Q-QAT findings that can then be discussed with the RCT CI to identify areas for targeted qualitative analysis. The technique is simple enough to be applied by a single researcher, although blind coding by another researcher increases the consistency and reliability of coding. It can be used as a stand-alone technique to identify potential recruitment barriers and provide recruiters with an easy overview of the recruitment process, although its real strength emerges when integrated with qualitative data analysis. Data from Q-QAT can be compared in various ways, for example, by centre, specialty, or particular recruiter.

## Discussion

This paper has presented the development, application and guidance for use of a new technique to rapidly identify recruitment issues in ongoing RCTs, particularly focusing on the presentation of information to potential participants by recruiters. The Quanti-Qualitative Appointment Timing (Q-QAT) technique incorporates simple quantitative techniques integrated with targeted qualitative approaches, allowing the rapid identification of potential difficulties and findings to aid understanding of underlying issues that can be addressed to improve RCT recruitment. The technique was developed in a programme of research employing primarily qualitative research methods to understand recruitment issues in six RCTs [9,11]. In this study the technique has been applied directly to two RCTs in different clinical contexts with recruitment difficulties of unknown origin.

Used as a stand-alone technique, Q-QAT rapidly identified weaknesses in information provision and potential recruitment barriers, such as minimal time spent explaining the RCT, and variations in time spent explaining the RCT treatments. Although these issues could only be fully understood when explored qualitatively, the initial patterns that emerged from the Q-QAT tables enabled ‘targeted’ qualitative analysis to facilitate a detailed and nuanced understanding of issues related to the presentation of the RCT. These combined findings were then presented to the RCT CI, so that a plan of action to improve recruitment practice could be initiated, including engaging recruiters in discussion of issues that are often hidden and uncomfortable, including aspects of equipoise and patient eligibility [13,14]. We presented the findings directly to the CI and then to individual recruiters and anonymously in group meetings. Feedback meetings can use the framework in Tables 2 and 3, and these tend to provoke considerable discussion. However, the particular process of feedback and discussion needs to be flexible in relation to the particular RCT and agreed with the RCT CI.

The idea for the technique emerged from a re-assessment of findings from qualitative research in previous RCTs [7,9,11]. Imbalanced information provision was a frequent finding and a key recruitment problem in these RCTs. While recognising the importance of routinely integrating fully fledged and comprehensive qualitative research into difficult RCTs [9], there was also a pressing need to identify simpler techniques aimed at rapidly understanding the reasons for difficulties that arise from the recruitment appointment. Ideally, such techniques should be applicable across a wide range of trial recruitment discussions, which can occur in the context of general clinical consultations or dedicated recruitment appointments. The Q-QAT technique was successful when applied to the two RCTs with recruitment difficulties described in this paper, which were selected to include simple and more complex recruitment pathways, an elective compared with life-threatening condition, and involving one clinical specialty or two. The technique received positive feedback from recruiters in both RCTs, and, when fully supported, helped improve recruitment in RCT2, as had an earlier informal version of the technique [7].

Time spent in discussion with patients has previously been considered in primary care settings, not in relation to the time spent on specific aspects of the discussion, but in relation to the total length of the consultation and its influence on aspects such as patient/physician satisfaction [16,17]. Increasing the length of the consultation does not always improve patient satisfaction, with the quality of the information provision found to be of greater importance [17,18]. RCTs are widely acknowledged as constituting the highest form of evidence to demonstrate the effectiveness of an intervention and yet the time spent by recruiters in discussing RCTs with patients has not previously been examined. The Q-QAT analysis quickly identified that very little time was spent during appointments on presenting or explaining the RCT, and that more time was needed to assist participant understanding. This could be achieved while retaining the same length of appointment by integrating the RCT discussion more effectively with the clinical issues, including mentioning the RCT earlier, and avoiding some repetition of treatment details.

There are some limitations to the study presented in this paper and the Q-QAT technique. The technique was developed during research with six RCTs and then applied formally only in two simple parallel-group treatment trials, and so it is possible that researchers applying the technique more widely might face different challenges to those described here. We attempted to address some of the limitations through the process of blind coding by three researchers to ensure that the six coding categories could be accurately, rapidly and consistently applied, and guidelines have been presented that standardise the technique. Although there were variations in the clinical context and complexity of the two RCTs presented in this study, the coding strategy was easily adapted to address these differences. The Q-QAT analysis required coding of audio-recordings or transcripts of appointments, which could then produce relatively rapid but somewhat crude quantitative findings identifying imbalances and time spent on the presentation of aspects of the RCT. Targeted qualitative research was then crucial to understand the quality of the information presented and its impact on recruitment.

Q-QAT was developed to be a technique that was specific enough to capture the amount of time spent discussing the key aspects of a RCT, but also sufficiently generic to be applied to RCTs of different designs and complexities. It is likely to be particularly useful for RCTs where recruitment is persistently difficult, even when other interventions have been tried. Q-QAT can be applied as a ‘trouble-shooting’ device when recruitment has been poor for some time, or as an integral part of a pilot or feasibility study where recruitment is predicted to be difficult - for example where there are marked differences between the arms, or one arm involves a very conservative management strategy compared with an interventional one, as in many RCTs involving surgery. The findings of the Q-QAT analysis might then inform aspects of the design or conduct of the main trial or identify recruiter training needs. In other cases, as in RCT2 here, it can provide information needed to enable an appropriate decision about whether or not to proceed with the transition to a large-scale RCT or further recruitment, providing essential information to convince a funder whether recruitment can or probably cannot be achieved.

The Q-QAT technique is now available as a stand-alone intervention to assist in understanding RCT recruitment and needs to be evaluated more thoroughly. Further work will be undertaken to evaluate the Q-QAT technique’s ability to identify training needs for RCT staff, as part of a wider, more complex intervention using mixed research methods to improve recruitment to RCTs while maintaining high levels of informed consent. This could include assessments of the quantity and quality of information between specialties or individual recruiters, as seen in RCT2. Further future work may consider the potential for the translation of the Q-QAT technique to other contexts, including as part of continuing monitoring of RCT recruitment efficiency and effectiveness, and perhaps within routine clinical practice in primary or secondary care.

## Conclusions

In conclusion, the Q-QAT technique is a novel and simple technique that can be used to identify and understand potential barriers to recruitment within RCT appointments. It can be used as a stand-alone technique to rapidly identify issues, but is more informative when followed by targeted qualitative analysis aimed at understanding the reasons for the patterns identified. The quanti-qualitative findings can then be discussed with RCT CIs, and used to develop strategies to improve recruitment and engage recruiters in discussing underlying issues that may be hidden or uncomfortable [13,14]. The technique’s ease of use and ability to provide useful insights into recruitment problems in a short time frame are key attributes that make it suitable for use in RCTs underway with recruitment difficulties and in feasibility/pilot studies developing RCTs with expected recruitment challenges.

## Abbreviations

CI: Chief Investigator
HTA: Health Technology Assessment
MDT: Multi-Disciplinary Team
MRC: Medical Research Council
NIHR: National Institute for Health Research
PI: Principal Investigator
ProtecT: Prostate testing for cancer and Treatment
Q-QAT: Quanti-Qualitative Appointment Timing
RCT: Randomised controlled trial
RfPB: Research for Patient Benefit
TTFMT: Time to the first mention of the RCT
